# Common variants in the genes of triglyceride and HDL-C metabolism lack association with coronary artery disease in the Pakistani subjects

**DOI:** 10.1186/s12944-017-0419-4

**Published:** 2017-01-31

**Authors:** Saleem Ullah Shahid, N.A. Shabana, Jackie A. Cooper, Abdul Rehman, Steve E. Humphries

**Affiliations:** 1 Department of Microbiology and Molecular Genetics, University of the Punjab, Lahore, Pakistan 54590; 2 Centre of Cardiovascular Genetics, British Heart Foundation Laboratories, University College London, London, WC1E6JF UK England

**Keywords:** SNPs, Triglycerides, HDL-C, KASPar

## Abstract

**Background:**

Serum Triglyceride (TG) and High Density Lipoprotein (HDL-C) levels are modifiable coronary artery disease (CAD) risk factors. Polymorphisms in the genes regulating TG and HDL-C levels contribute to the development of CAD. The objective of the current study was to investigate the effect of four such single nucleotide polymorphism (SNPs) in the genes for Lipoprotein Lipase (*LPL)* (rs328, rs1801177), Apolipoprotein A5 (*APOA5)* (rs66279) and Cholesteryl ester transfer protein (*CETP)* (rs708272) on HDL-C and TG levels and to examine the association of these SNPs with CAD risk.

**Methods:**

A total of 640 subjects (415 cases, 225 controls) were enrolled in the study. The SNPs were genotyped by KASPar allelic discrimination technique. Serum HDL-C and TG were determined by spectrophotometric methods.

**Results:**

The population under study was in Hardy Weinberg equilibrium and minor allele of SNP rs1801177 was completely absent in the studied subjects. The SNPs were association with TG and HDL-C levels was checked through regression analysis. For rs328, the effect size of each risk allele on TG and HDL-C (mmol/l) was 0.16(0.08) and −0.11(0.05) respectively. Similarly, the effect size of rs662799 for TG and HDL-C was 0.12(0.06) and −0.13(0.0.3) and that of rs708272 was 0.08(0.04) and 0.1(0.03) respectively. The risk allele frequencies of the SNPs were higher in cases than controls, but the difference was not significant (*p* > 0.05) and SNPs were not associated with CAD risk (*p* > 0.05). The combined gene score of four SNPs significantly raised TG and lowered HDL-C but did not increase CAD risk.

**Conclusion:**

The studied SNPs were associated with TG and HDL-C levels, but not with CAD in Pakistani population under study.

**Electronic supplementary material:**

The online version of this article (doi:10.1186/s12944-017-0419-4) contains supplementary material, which is available to authorized users.

## Background

Lipoprotein lipase (LPL), a 61 kDa heparin releasable enzyme is attached to capillary endothelium at glycosaminoglycan residues [1–3]. It is a rate limiting enzyme in hydrolysis of triglycerides (TG) from core of chylomicrons (CM) and very low density lipoproteins (VLDL) into free fatty acids and glycerol generating CM remnants from CM, intermediate density lipoprotein (IDL) from VLDL and eventually low density lipoprotein (LDL) cholesterol by the action of hepatic lipase. Phospholipids and surface free cholesterol are converted to HDL-C [4]. Decreased LPL activity is associated with increased TG and decreased HDL-C levels and accumulation of atherogenic TG rich lipoproteins in circulation [5–8]. Although affected by environmental factors and life style, genetic variants within a population may also influence TG and HDL-C levels. It has been estimated that heritability accounts for >30% hypertriglyceridemia cases [9].

The *LPL* gene is highly polymorphic and more than 100 variants have been identified in it [10]. The common variants with modest effect on TG and HDL-C levels may prove to be an important CAD risk contributors at population levels. The polymorphism *LPL* rs328 is a C > G change in exon nine and changes the codon for serine 447 (TCA) to TGA which is a stop codon hence also given the name Ser447Stop or S447X. This results in pre-mature truncation of the protein which is short of two amino acids. The product of LPLS447X is missing C terminal serine and glycine residues and also generates restriction site for the enzyme *Mnl*I [11]. *LPL* rs1801177 is a mis-sense polymorphism and causes substitution of aspartic acid (D) to asparagine (N) hence also denoted as Asp9Asn or D9N.

Apo-lipoprotein A5 (ApoA5) is part of VLDL, HDL and CM and is a major regulator of blood TG and HDL-C through interaction with LDLR [12]. ApoA5 controls TG levels directly by increasing the breakdown of TG rich lipoproteins through the action of LPL and indirectly by reducing VLDL biosynthesis [13]. The expression of *ApoA5* is in inverse relation with circulating plasma TG levels. *ApoA5* knockout mice had 4 folds higher TG concentration and when knocked in with human *ApoA5*, it caused a 50-70% reduction in mouse serum TG level [14]. The *APOA5* rs662799 is the most studied SNP in the promoter region of this gene [15]. Different allele frequencies of this polymorphism have been reported in Chinese and Europeans [16] and is the most effective SNP in *ApoA5* gene [17]. The SNP is associated with decreased ApoA5 levels and high plasma TG levels and the association has been shown to be independent of other factors.

Cholesteryl ester transfer protein (CETP) transfers lipids between lipoproteins and redistributes triglycerides, cholesteryl esters and phospholipids between lipoproteins [18, 19]. An inverse correlation exists between CETP activity and blood HDL-C levels [20]. Many functional polymorphisms have been identified in *CETP* gene [21]. The association of *CETP* with HDL-C is stronger than any other locus in the genome [22]. The polymorphism *CETP* rs708272 also known as *Taq1B* polymorphism in this gene is the most studied one. The effect of this SNP is also modulated by the factors like smoking and alcohol consumption [21].

The effect of these common variants in the genes for lipid metabolism on blood lipid levels has been studied well in Caucasians. However, the data on Pakistani people is limited. As is known that the allele frequencies and linkage disequilibrium of a SNP with tag/functional SNP may vary among ethnicities, the authors of the study hypothesized that the SNPs in the above mentioned genes may have strong effect on blood TG and HDL-C levels in Pakistani population which is facing high CAD burden. In this study, we examined the effect of polymorphisms *LPL* rs328, *LPL* rs1801177, *ApoA5* rs662799 and *CETP* rs708272 on serum TG and HDL-C levels and their association with CAD. For this purpose, a case control study was opted. As the common variants individually have modest effects, the cumulative effect of the four SNPs was examined by combining them in a small gene score.

## Methods

### Study subjects

A total of 640 subjects including 415 CAD cases and 225 healthy controls were enrolled in the study. The inclusion criteria for selection of cases and controls has been already described [23]. The CAD patients were selected from tertiary care hospitals of the province of Punjab, Pakistan. These were diagnosed cases based upon the ECG, cardiac echo, radiologic and troponine T/I levels. These cases were recently diagnosed of having CAD and were not taking any lipid lowering or antihypertensive drug. Control subjects were apparently healthy individuals from the general population with BMI 18.5-22.99 Kg/m^2^ and no history of heart disease. The exclusion criteria included the presence of any chronic ailment like chronic liver or kidney disease, cancers or any ongoing acute infection or disease and CAD patients with obesity. The participants gave a written informed consent and the study was approved by the institutional ethical committee, university of the Punjab, Lahore.

### Biochemical and genetic analysis

Serum TG and HDL-C were measured using commercially available kits (Human diagnostics, Germany). A 96 well plate reader, Epoch (Biotek instruments, Highland Park) was used for end stage spectrophotometric measurements. The DNA was extracted from whole blood leucocytes using commercially available genomic DNA purification kit (Promega Wizard, USA). The DNA was quantified using nano drop (ND-8000, USA). The DNA samples were arrayed into 384 well plates (Micro Amp) by an automated robotic liquid handling system (Biomerk FX, Beckman Coulter). The SNPs were genotyped by florescence based allelic discrimination technique KASPar, the details described elsewhere [24]. After amplification, the results were analysed on ABI Prism 7900HT (Applied Biosystems/Life Technologies) and the genotypes were called by sequence detection software (SDS, version 2.0). The sequence of primers and probes used for KASPar are given in Additional file 1: Table S1. The genotyping results were also confirmed by direct DNA sequencing (Source Biosciences, UK) and the results were always same. The DNA fragment containing polymorphism was amplified using commercially available master mix (Qiagen). The sequence of primers used for direct DNA sequencing is given in Additional file 2: Table S2. The genotyping quality was also checked by the inclusion of non-template controls and known heterozygotes in the test plate.

### Statistical analysis

The results were analysed using statistical package for social sciences (SPSS), IBM version 22.0. Independent sample *t* test was used to compare continuous variables between cases and controls. Hardy-Weinberg equilibrium of genotypes in studied subjects was assessed by a chi squared test. The allele frequencies were compared between cases and controls by chi squared test [25]. The association of SNPs with TG and HDL-C was estimated by linear regression. As CAD is a binary variable, the association of SNPs with CAD was calculated by binary logistic regression. One way analysis of variance (ANOVA) was used to calculate mean TG and HDL-C levels along different genotypes. The effect size (β effect) which is per risk allele increase/decrease in TG and HDL-C levels was calculated by linear regression and indicated by β with standard error (Se). Differences in regression slope between groups were tested by fitting an interaction term in the regression model. The relationship between gene score, frequency of individuals with a particular gene score and TG and HDL-C was plotted using an excel spread sheet. For all measurements, statistically significant cut-off was adjusted at a *p*-value <0.05. An un-weighted gene score of four SNPs was calculated by summing up the number of risk alleles at all the four loci.

## Results

The baseline biochemical and anthropometric parameters of the subjects under study are given in Table 1. Serum TG and HDL-C were significantly higher in the cases than the controls. The CAD cases were more hypertensive, diabetic, and smoking rate was also significantly higher than controls. The genotyping rate for all the SNPs was >95%. The basic features of the study SNPs and the Hardy Weinberg equilibrium *p*-values are provided in the Additional file 3: Table S3. The study population was in Hardy Weinberg equilibrium collectively as well as individually among cases and controls except *LPL* rs1801177, for which a quite monomorphic picture was observed and risk allele was absent in the subjects studied. In the case of rs328, rs662799 and rs708272, the RAFs were higher in the cases than the controls, but the difference was not statistically significant as the chi squared *p* values were >0.05. A mean gene score of four SNPs was also not significantly higher in the cases than the controls (Table 2). The SNPs under study were not associated with CAD outcome. The odds ratio (OR) of all the SNPs was higher in the cases than the controls, but was not statistically significant. The combined gene score of 4 SNPs was also not associated with CAD (Table 3).

**Table 1:** Anthropometric and biochemical features of the subjects under study

Variables	Cases	Controls	*p*-value
Sample number	415	225	-
Age (years)	59.1 ± 12.6	56 ± 10.5	0.002
Gender			
Males (n)	222	123	0.27
Females (n)	193	102	
Diabetes (%)	64.6	13.6	5.1 × 10^−34^
Hypertension (%)	62.1	16.4	8.9 × 10^−28^
Smoking (%)	29.5	10.5	7.3 × 10^−08^
Total cholesterole (mmol/L)	5.37	4.54	7.8 × 10^−13^
Triglycerides (mmol/L)	2.4	2.12	2.3 × 10-5
HDL-C (mmol/L)	1.17	1.74	1.5 × 10^−65^
LDL-C (mmol/L)	2.74	2.19	6.1 × 10^−21^

**Table 2:** Comparison of RAFs between cases and controls

SNP	Alleles	RAF		**p-value
		Cases	Controls	
rs328	*C/G	0.94 (0.92-0.95)	0.91 (0.88-0.93)	0.060
rs1801177	G/A*	0	0	-
rs662799	A/G*	0.170 (0.14-0.20)	0.167 (0.13-0.20)	0.89
rs708272	*C/T	0.551 (0.52-0.59)	0.543 (0.50-0.59)	0.80
Mean gene score ± SD		3.31 ± 0.94	3.23 ± 0.96	0.319

*is risk allele, **Chi squared *p*-value

**Table 3:** Association of the SNPs and gene score with CAD

SNP	OR	C.I	*p*-value
rs662799	1.02	0.75–1.4	0.9
rs708272	1.03	0.82–1.3	0.81
rs328	1.5	0.98–2.3	0.06
rs1801177	–	–	–
Gene score	1.09	0.92–1.3	0.32

*OR* Odds ratio, *C.I.* Confidence interval

### Association of the selected SNPs with serum triglyceride and high density lipoprotein cholesterol levels

The effect of SNPs on TG and HDL-C levels was examined individually. The risk alleles of all the SNPs raised TG levels, but the β effect was only marginally significant. The risk alleles significantly lowered HDL-C levels in the subjects under study as shown by their β effect *p* values <0.05 (Table 4). Individually, each risk allele increased TG and decreased HDL-C and the combined gene score was significantly associated with both TG and HDL-C levels (Table 5).

**Table 4:** Mean TG and HDL-C levels according to genotypes

SNP	Genotype	TG(mmol/L)		HDL-C(mmol/L)	
		Mean TG	95% C.I	Mean HDL-C	95% C.I
rs328	GG	1.93 ± 0.14	1.7–2.1	1.8 ± 0.17	1.5–2
	GC	2.2 ± 0.71	2–2.3	1.44 ± 0.47	1.3–1.5
	CC	2.3 ± 0.8	2.3–2.4	1.36 ± 0.44	1.3–1.4
	β (se)	0.16(0.08)		−0.11(0.05)	
	R2	0.2%		1.1%	
	*p*-value	0.06		0.02	
rs662799	AA	2.27 ± 0.77	2.2–2.3	1.42 ± 0.45	1.4–1.5
	AG	2.36 ± 0.83	2.2–2.5	1.28 ± 0.42	1.2–1.3
	GG	2.56 ± 0.61	2.3–2.9	1.2 ± 0.41	1–1.4
	β (se)	0.12(0.06)		−0.13(0.03)	
	R2	0.6%		0.8%	
	*p-*value	0.053		<0.0001	0.001
rs708272	TT	2.22 ± 0.7	2.1–2.3	1.5 ± 0.42	1.4–1.6
	CT	2.3 ± 0.8	2.2–2.4	1.4 ± 0.5	1.3–1.4
	CC	2.4 ± 0.8	2.3–2.5	1.3 ± 0.42–	1.2–1.3
	β (se)	0.08(0.04)		0.1(0.03)	
	R2	0.1%		1.9%	
	*p*-value	0.08		<0.001	

*β* (*Se*) is per risk allele increase/decrease in lipid trait (Standard error)

**Table 5:** Correlation of gene score with TG and HDL-C

Gene score	Correlation	TG	HDL-C
Unweighted	Correlation	r = 0.127	r = −0.246
	β (se)	0.127	−0.246
	*p*-value	0.001	<0.0001

### Quantitative effect of SNPs on the serum parameters

**Fig. 1:**
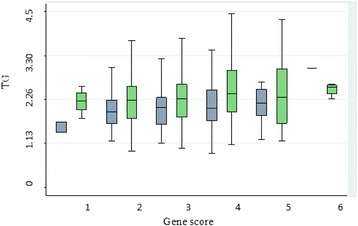
*Green bars* represent cases and *grey bars* represent controls

**Fig. 2:**
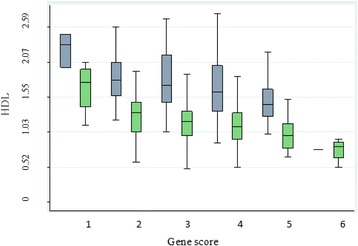
*Green bars* represent cases and *grey bars* represent controls

The mean increase in TG and a decrease in HDL-C with the increasing number of risk alleles and the difference in TG and HDL-C levels between those having lowest and highest number of risk alleles was measured. It was evident that TG was significantly higher and HDL-C was significantly lower in subjects having highest number of risk allele than those having lowest number of risk alleles (Table 6). The correlation between TG/HDL-C, gene score and CAD was also examined. It was clear that more CAD cases showed skewed distribution towards the high gene score and TG levels. Whereas, more controls were shifted towards the left side of the normal distribution curve with lower gene score and TG levels. Contrarily to TG levels, as low HDL-C is a CAD risk factor, a reverse pattern was observed. The control subjects having higher HDL-C were concentrated towards the left side, whereas more cases were located towards the right side of the graph where the gene score was high (Figs. 1 and 2).

**Table 6:** The effect of increasing risk allele on TG and HDL-C

Gene score	TG	HDL-C
	(Δ)	(Δ)
2	0.24	−0.44
3	0.05	−0.1
4	0.2	−0.08
5	0.06	−0.13
6	0.25	−0.41
^a^Δ	0.69	−1.16

Δ is change in TG and HDL-C levels with the addition of each risk allele in gene score and ^a^Δ is the difference in TG and HDL-C between those having maximum and those having minimum number of risk alleles

## Discussion

The prevalence of coronary artery disease has increased globally, however, the larger proportion of the burden is shared by the developing countries due to larger populations. As a complex disorder, it is difficult to dissect the genetic and environmental components involved in the progression of the disease. The unique social and cultural practices prevailing in the Pakistani population tend to concentrate the risk alleles predisposing to the heart diseases. A large number of genes have been identified to be associated with the risk of cardiac diseases, however, among these the genes involved in the synthesis and metabolism of serum lipids have been subject of intense research due to their clinical significance and their ability to be quantified from the serum. We therefore selected variants in the genes that affect two key serum lipids namely triglycerides and high density lipoprotein cholesterol with the aim to check whether any change in the genes results in the change of the serum levels of these lipids.

The association of *LPL* polymorphisms with lipid levels has been controversial [6]. Most of the SNPs studied in this gene are rare and limited either to single families or are restricted to isolated geographic regions, whereas, common variants show differences in allele frequencies among different populations [26]. In our study, minor allele (G) of the SNP *LPL* rs328 was found to be protective as its presence lowers TG and raises HDL-C. The common allele (C) was the risk allele. The carriers of risk allele had higher plasma TG and lower HDL-C levels. However, a significant difference in allele frequencies was not observed in cases than controls. Since the *LPL* rs328 causes a protein change towards the C terminal, hence it affects the legend function of the protein leading to enhanced uptake of lipoproteins at cellular receptors [27]. Hence this is a gain in function change and such changes are rare in nature and prevent from cardiovascular events [28]. Such mutations are blessing in present eras where people are living with a large number of CAD risk factors. Our results were also inconsistent with many other studies [29–33]. The risk allele of *LPL* rs1801177 was completely absent in the subjects under study. In European people, the frequency of this allele has been reported to vary from 1.6 – 6.7% [34]. In another meta-analysis of 89 studies, the average frequency of risk allele was less than 3% ranging from <0.1 – 8% [10]. We previously have reported the RAF of 0.01% in another study group from Pakistan [35].

We have replicated the effect of *ApoA5* rs662799 in Pakistani subjects and similar results were reported in a study conducted in India where the RAF was associated with 19% increase in serum TG levels [36]. Similar TG raising effect of this SNP has also been reported in Chinese people [15, 17]. Since the allele frequencies of this SNP show interethnic variations [37], its association with biochemical markers may also vary in different ethnicities. Due to the presence of protective variants, black population whether from Caribbean or South African ancestries have low TG, high HDL-C with less CAD mortality than the white populations. Whereas South Asian immigrants to Europe exhibit high mortality rate associated with CAD secondary to raised serum TG and HDL-C [38]. Similar effect of this variant on blood lipids have been documented in Caucasians, African American and Hispanics [16, 39–41].

The presence of C allele of *CETP* rs708272 decreased HDL-C and increased TG levels, both are CAD risk factors. The results were in agreement with another prospective study where each C allele was associated with 3.1 mg/dl decrease in HDL-C concentration and a 24% increase in CAD risk [42]. In a meta-analysis of 98 published studies, the protective allele T was associated with lower CETP mass and activity and higher mean HDL-C levels [43]. Similar results have also been reported in Chinese people [44]. The role of CETP protein in lipid metabolism, though largely studied, is still controversial. According to Thompson et al. (2008), the *CETP* polymorphisms were associated with moderate inhibition of CETP activity, increased HDL-C concentration and decreasing CAD risk. However, some other studies have reported greater CAD risk associated with decreased CETP concentration [45, 46]. Furthermore, the effect of this SNP is also modulated by environmental effects [21] and the LD pattern of the tag SNPs in *CETP* gene is also different in Asians compared to Europeans [20].

The study had some limitations, it included only four polymorphisms in the lipid metabolism genes, inclusion of other polymorphisms in a large number of genes affecting serum lipid traits may have improved the results of the study and should be considered in future. Furthermore, the study had relatively small sample size therefore future studies with even larger sample size and more variants included, should be done in order to confirm the findings of the current study. The implications of the study include providing the baseline information about the polymorphisms in the Pakistani population and the probable mechanism through which these variants may affect TG and HDL-metabolism.

## Conclusion

In conclusion, the SNPs were associated with blood TG and HDL-C levels additively but we could not find a significant high risk allele frequency in the cases than the controls. However, because the SNPs had small effect size and association with CAD was also modest, we tried to examine their combined effect on CAD risk and TG and HDL-C levels. The association with CAD still remained robust; however, the combined effect of the SNPs was highly significant on TG and HDL-C levels. This information can be used to establish panels comprising of common variants with modest effect on the serum lipid traits to establish protocols for identifying the genetic component responsible for changes in serum lipid chemistry in a routine clinical setting.

## Additional files

Additional file 1: Table S1. List of primers and probes used in KASPar assay. (DOCX 11 kb)
Additional file 2: Table S2. Sequence of primers used in PCR. (DOCX 11 kb)
Additional file 3: Table S3. Basic features of SNPs under study. (DOCX 13 kb)

## Abbreviations

ANOVA: Analysis of variance
CAD: Coronary artery disease
CETP: Cholesterol ester transfer protein
CM: Chylomicrons
HDL-C: High density lipoprotein cholesterol
IDL: Intermediate density lipoprotein
LDL: Low density lipoprotein
LPL: Lipoprotein lipase
OR: Odds ratio
SDS: Sequence detection software
SNPs: Single nucleotide polymorphisms
SPSS: Statistical package for social sciences
TG: Triglycerides
