# Supplementary material for: Common variants in the genes of triglyceride and HDL-C metabolism lack association with coronary artery disease in the Pakistani subjects
Source: Lipids Health Dis. 2017 Jan 31;16:24. doi: 10.1186/s12944-017-0419-4 (PMC5282842; doi:10.1186/s12944-017-0419-4)
Supplement: Additional file 1: Table S1. — List of primers and probes used in KASPar assay. (DOCX 11 kb) [file 12944_2017_419_MOESM1_ESM.docx]

**Supplementary table 1**: **List of primers and probes used in KASPar assay**

| Gene | SNP | Sequence ( 50bp either side, allele label = [A/T], Other SNPs = N or IUPAC Code ) | FAM | VIC |
| --- | --- | --- | --- | --- |
| *CETP* | rs708272 | TTTACCCCCTGACTCAACCCCCTAACCTGGCTCAGATCTGAACCCTAACT[**C/T]**GAACCCCANTGATTCTGGGTCTCAGACAAACACAAATCCCTATACCTGGC | C | T |
| *APOA5* | rs662799 | AAGAGGCATCTGGGCCAGNGACTCTGAGCCCCAGGAACTGGAGCGAAAGT**[A/G]**AGATTTGCCCCATGAGGAAAAGCTGAACTCCACTCGCAGGGCCTCTGAGG | A | G |
| *LPL* | rs328 | GGCACCTGCGGTATTTGTGAAATGCCATGACAAGTCTCTGAATAAGAAGT**[C/G]**AGGCTGGTGAGCATTCTGGGCTAAAGCTGACTGGGCATCCTGAGCTTGCA | C | G |
| *LPL* | rs1801177 | CAGTTAACCTCATATCYAATTTTTCCKTTCCAGAAAGAAGAGATTTTATY**[G/A]**ACATYGRAAGTAAATTTGCCCTAAGGAMCCCTGAAGWCACAGSTGARGAC | G | A |
