# Supplementary material for: Common variants in the genes of triglyceride and HDL-C metabolism lack association with coronary artery disease in the Pakistani subjects
Source: Lipids Health Dis. 2017 Jan 31;16:24. doi: 10.1186/s12944-017-0419-4 (PMC5282842; doi:10.1186/s12944-017-0419-4)
Supplement: Additional file 2: Table S2. — Sequence of primers used in PCR. (DOCX 11 kb) [file 12944_2017_419_MOESM2_ESM.docx]

**Supplementary table 2**: **Sequence of primers used in PCR**

| Primer | Sequence |
| --- | --- |
| *CETP* rs708272*_*F | 5'-GTGACCCCCAACACCAAATA-3' |
| *CETP* rs708272_R | 5'-TCGCCTTCAAGGTCAAGTTC-3' |
| *APOA5* rs662799_F | 5'-GCAGGGTGAAGATGAGATGG-3' |
| A*POA5* rs662799_R | 5'-TAGACGGAGTGGGTGTGTCA-3' |
| *LPL* rs328_F | 5'-CTTCCACAGGGTGATCTTCTG-3' |
| *LPL* rs328_R | 5'-CATGAAGCTGCCTCCCTTAG-3' |
| *LPL* rs1801177_F | 5'-AAATAGCATCAGCGGTGGTT-3' |
| *LPL* rs1801177_R | 5'-ATGAGGTGGCAAGTGTCCTC-3' |
