# Supplementary material for: Common variants in the genes of triglyceride and HDL-C metabolism lack association with coronary artery disease in the Pakistani subjects
Source: Lipids Health Dis. 2017 Jan 31;16:24. doi: 10.1186/s12944-017-0419-4 (PMC5282842; doi:10.1186/s12944-017-0419-4)
Supplement: Additional file 3: Table S3. — Basic features of SNPs under study. (DOCX 13 kb) [file 12944_2017_419_MOESM3_ESM.docx]

**Supplementary** **Table 3: Basic features of SNPs under study**

| Gene | CHR | SNP | Call rate % | HWE-*p* | | |
| --- | --- | --- | --- | --- | --- | --- |
|  |  |  |  | Cases | Controls | Total |
| *LPL* | 8p21 | rs328 | 96 | 0.58 | 0.39 | 0.76 |
| *LPL* | 8p21 | rs1801177 | 97 | - | - | - |
| *APOA5* | 11q23 | rs662799 | 96 | 0.57 | 0.35 | 0.93 |
| *CETP* | 16q13 | rs708272 | 97 | 0.27 | 0.65 | 0.54 |

HWE-*p* = Hardy Weinberg equilibrium significance value.
